# The physics of radioembolization

**DOI:** 10.1186/s40658-018-0221-z

**Published:** 2018-11-02

**Authors:** Remco Bastiaannet, S. Cheenu Kappadath, Britt Kunnen, Arthur J. A. T. Braat, Marnix G. E. H. Lam, Hugo W. A. M. de Jong

**Affiliations:** 1 Department of Radiology and Nuclear Medicine, University Medical Center Utrecht, Room E01.132, P.O. Box 85500, 3508 GA Utrecht, The Netherlands; 2 Department of Imaging Physics, The University of Texas MD Anderson Cancer Center, 1155 Pressler St, Unit 1352, Houston, TX 77030 USA

**Keywords:** Radioembolization, Personalized medicine, Dosimetry, Radiobiological model, Dose-effect relationship, Theranostics

## Abstract

Radioembolization is an established treatment for chemoresistant and unresectable liver cancers. Currently, treatment planning is often based on semi-empirical methods, which yield acceptable toxicity profiles and have enabled the large-scale application in a palliative setting. However, recently, five large randomized controlled trials using resin microspheres failed to demonstrate a significant improvement in either progression-free survival or overall survival in both hepatocellular carcinoma and metastatic colorectal cancer. One reason for this might be that the activity prescription methods used in these studies are suboptimal for many patients.

In this review, the current dosimetric methods and their caveats are evaluated. Furthermore, the current state-of-the-art of image-guided dosimetry and advanced radiobiological modeling is reviewed from a physics’ perspective. The current literature is explored for the observation of robust dose-response relationships followed by an overview of recent advancements in quantitative image reconstruction in relation to image-guided dosimetry.

This review is concluded with a discussion on areas where further research is necessary in order to arrive at a personalized treatment method that provides optimal tumor control and is clinically feasible.

## Background

Radioembolization is an established treatment for chemoresistant and unresectable liver cancers. The treatment consists of the administration of microspheres that are loaded with a beta-emitter into the arterial hepatic vasculature. As a result of a differential vasculature of the healthy liver and tumor tissue, the microspheres preferentially accumulate in the tumor tissue, resulting in a local radiation dose to the tumor whilst sparing healthy liver tissue.

Currently, two types of microspheres are approved for clinical use by the FDA and are CE-marked: resin microspheres (SIR-spheres; SirTex Medical) and glass microspheres (TheraSphere; BTG International Ltd.), both of which are loaded with ^90^Y. A third type consists of ^166^Ho-loaded poly-lactate spheres, called QuiremSpheres, which is yet to receive FDA approval but has been CE-marked.

Radioembolization treatment planning is currently based on semi-empirical methods, which are designed to yield acceptable toxicity profiles and have enabled the large-scale application in a palliative setting. The addition of radioembolization with SIR-spheres to first-line treatments for metastatic colorectal cancer was investigated in three large randomized controlled trials, SIRFLOX [1], FOXFIRE [2], and FOXFIRE-global. The combined analyses of these three trials did not show a significant improvement in either progression-free survival [3] or overall survival [4]. Similarly, the SARAH and SIRveNIB Phase III studies failed to show an improvement in overall or progression-free survival after the treatment of advanced hepatocellular carcinoma with SIR-spheres vs. sorafenib [5, 6].

One reason for this might be that the current activity planning methods often result in underdosing (and in some cases overdosing) in patients [1, 2, 7–9]. Fortunately, a recent survey amongst European institutes has shown that some form of absorbed dose-based prescription was used by 64 and 96% of the respondents for the use of resin and glass microspheres, respectively [10]. The lack of biological clearance of the microspheres simplifies dosimetry compared to most other molecular radiotherapies. In order to further increase the adoption of absorbed dose-based prescription, the package inserts of both manufacturers could be improved by placing more emphasis on this type of activity prescription. Furthermore, there is mounting evidence for clear dose-effect relationships (see Table 1). However, the estimated absorbed dose needed to elicit a reliable tumor response or complication varies between studies. As such, reliable absorbed dose targets and limits are yet to be established.

**Table 1:** Non-exhaustive overview of recent dose-response studies showing a large variety in all relevant parameters. This variety in outcome measures and reported dose thresholds complicate data pooling and the extraction of reliable clinical dose limits

Study	Tumor type	Microsphere	Modality	Outcome	Dose model	Tumor dose	Liver dose	Remarks
Chiesa [61]	HCC	Glass	^99m^Tc-MAA SPECT	Choi 50% (CR and PR) [64]	EUD, BED, EUBED, *D*_avg_	TCP(50%) 560 Gy (*D*_avg_)	NTCP(50%) 97 Gy (*D*_avg_)	No scatter correction; dose reported here based on SPECT-based delineation; large influence of tumor volume
Srinivas [58]	HCC	Glass	^90^Y PET	mRECIST (CR, PR)	*D*_avg_	*D*_avg_ responders = 215 Gy	–	Non-significant association
Garin [135, 136]	HCC	Glass	^99m^Tc-MAA SPECT	EASL (CR, PR and SD)	*D*_avg_	Threshold for response = 205 Gy	84 Gy	Large, heterogeneous tumors probably require higher dose.
Chan [137]	HCC	Glass	^90^Y PET	mRECIST	*D*_median_, *D*_70_	*D*_median_ responders = 225 Gy; *D*_median_ non-responders = 83 Gy; *D*_70_ responders = 140 Gy; *D*_70_ non-responders = 24 Gy	–	
Kappadath [134]	HCC	Glass	bSPECT	mRECIST	*D*_avg_, BED_avg_	TCP (50%) *D*_avg_ = 160 Gy (95% CI = 123 to 196 Gy)*D*_median_ responders: 209 Gy, non-responders: 138 Gy	No complication observed for normal liver *D*_avg_ < 44 Gy	
Fowler [138]	HCC, NET, CRC	Resin and glass	^90^Y PET/MR	(v)RECIST	*D*_20_, *D*_70_, *D*_avg_	*D*_avg_ (29.8 Gy; sensitivity 76.9%; specificity 75.9%) and *D*_70_ (42.3 Gy; sensitivity 61.5%; specificity 96.6%) were predictive of response in CRC; No link found for other tumor types	–	
Strigari [68]	HCC	Resin	bSPECT	EASL/RECIST	BED	TCP(50%) 110–120 Gy	NTCP (50%) 52 Gy	
Kao [54]	HCC and cholangio	Resin	^90^Y PET	mRECIST + ‘minor response’	*D*_70_	*D*_70_ > 100 Gy (HCC); *D*_70_ > 90 Gy (cholangio)	–	Only patients with TN > =2 on MAA SPECT were selected for this study
Flamen [57, 139]	CRC	Resin	^99m^Tc-MAA SPECT	TLG change > 50%	*D*_avg_	*D*_avg_ > = 66 Gy (c.i. 32–159 Gy)	–	Chang’s attenuation correction Patient-relative calibration; assuming no LSF
Van der Hoven [140]	CRC	Resin	^90^Y PET	TLG change > 50%	*D*_avg_ and LMER	*D*_avg_ 40–60 Gy minimally	–	Higher baseline TLG leads to a higher reduction
Willowson [59]	CRC	Resin	^90^Y PET	TLG change > 50%	*D*_avg_	50 Gy	–	At lower doses, heterogeneity becomes more important
Eaton [55]	Metastatic melanoma	Resin	bSPECT	TLG change and SUV_max_	*D*_max_, *D*_avg_, *V*_50_	Sign. associations *D*_max_ with decreased TLG; *D*_avg_ and *V*_50_ with an absolute decrease in SUV_max_. Stronger effect for *D* > 50 Gy	–	No scatter or attenuation correction. 12 mm post-filter; crystal effects neglected
Chansanti [141]	NET	Resin	^99m^Tc-MAA SPECT	mRECIST	*D*_avg_	*D*_avg_ responders = 285.8 Gy; D_avg_ non-responders = 128.1 Gy	Patients with moderate to severe toxicity received *D*_avg_ > 50 Gy on the liver	Reported response at early (median 2.3 months) follow-up

*HCC* hepatocellular carcinoma, *NET* neuroendocrine tumor, *CRC* colorectal cancer, *Cholangio* cholangiocarcinoma, *LMER* linear mixed-effects regression model, *CR* complete response, *PR* partial response, *SD* stable disease

This review aims to investigate the current state-of-the-art of dosimetry in relation to this discussion from a physics perspective, elaborating on technical difficulties and providing an overview of the relevant hiatuses in the current knowledge.

## Review

### Current activity planning methods

#### Pre-treatment safety procedure

Before the infusion of the therapeutic dose, an angiographic work-up is performed in which the hepatic vessel anatomy is explored and an infusion site is selected. As per EANM guidelines, this is followed by the administration of 75–150 MBq of the surrogate particle ^99m^Tc macroaggregated albumin (^99m^Tc-MAA) [11]. These imageable protein aggregations aim to simulate the expected distribution of the subsequent therapeutic microspheres. There are three main reasons for the use of a simulation procedure using ^99m^Tc-MAA [12].

First, extrahepatic depositions can be detected. This used to be done using planar scintigraphy; however, SPECT/CT has been shown to be superior for this goal [13].

Second, the lung shunt fraction (LSF) is estimated. This fraction is used as a proxy for the absorbed lung dose and is subsequently used to adjust the prescribed activity, as described in the next sections. The microsphere manufacturers specify that this estimation should be performed on planar scintigraphic imaging [14], according to the formula

1 $$ \mathrm{LSF}=\frac{C_{\mathrm{lungs}}}{C_{\mathrm{lungs}}+{C}_{\mathrm{liver}}}, $$

where *C*_lungs_ indicates the total counts in the lungs, and *C*_liver_ the total counts in the liver. Usually, the number of counts in these regions-of-interest (ROIs) is determined on the geometric mean of the anterior and posterior views. However, the validity method has been questioned as it does not include proper compensation for differences in attenuation between the liver and the lungs, resulting in a systematic overestimation of LSF estimated on planar images relative to LSF estimated on SPECT/CT images [15–17].

Third, by using the ^99m^Tc-MAA distribution as a predictor for the subsequent ^90^Y distribution, it may be used for multi-compartment dosimetry (see the section “Multi-compartment dosimetry”) [18].

The type of planning method used in clinical practice depends on the type of microsphere. For resin microspheres, the most commonly used method is the body surface area-based (BSA) method [14]. For glass microspheres and holmium-loaded microspheres, a commonly used method is the MIRD mono-compartment method [19–21]. Collectively, these methods are referred to as semi-empirical methods.

#### BSA-based method for resin microspheres

The BSA-based method was developed to overcome the clinically observed high toxicity of a previous method used in early clinical studies [22]. The prescribed activity using this previous method ranged between 2 and 3 GBq, depending on tumor load only and not on the liver size [14]. Conversely, the BSA-based method is based on the observation that BSA correlates with liver volume in the healthy population [23]. As such, the planned activity is adjusted to an individual patient’s liver volume. The activity is calculated according to the following relationship [14]:

2 $$ A\left[\mathrm{GBq}\right]=\left(\mathrm{BSA}\left[{m}^2\right]-0.2\right)+\frac{V_{\mathrm{tumor}}}{V_{\mathrm{tumor}}+{V}_{\mathrm{normal}\ \mathrm{liver}}}, $$

where *V*_tumor_ and *V*_normal liver_ indicate the volumes of the tumor and the healthy parenchyma, respectively. For lobar or superselective treatment, the activity is reduced in proportion to the size of the liver volume being treated.

The prescribed activity is reduced by 20 or 40% if there is an LSF between 10 and 15% or 15 and 20%, respectively. An LSF higher than 20% is a contraindication for the treatment [14].

A modified BSA method was used for the SIRFLOX, FOXFIRE, and FOXFIRE-global studies, where activity was reduced relative to the BSA method, based on LSF and tumor involvement [1].

#### MIRD mono-compartment for glass microspheres

For glass microspheres, the activity calculation is based on the desired mean absorbed dose to the target liver mass (independent of tumor burden), following:

3 $$ A\left[\mathrm{GBq}\right]=\frac{\mathrm{Desired}\ \mathrm{dose}\ \left[\mathrm{Gy}\right]\times {M}_{\mathrm{target}}\left[\mathrm{kg}\right]}{50\ \left[\mathrm{J}/\mathrm{GBq}\right]}. $$

The desired absorbed dose is set assuming a completely homogeneous distribution of the microspheres over the target volume. The target mass may be determined using either CT, MRI, PET, or ^99m^Tc-MAA SPECT [21].

The recommended absorbed dose ranges from 80 to 150 Gy, depending on the judgment of the treating physician. The estimated total activity shunting to the lungs should not exceed 610 MBq, which equates to approximately 30 Gy in 1 kg lung tissue [21].

#### MIRD mono-compartment method for holmium microspheres

For the administration of holmium microspheres, a methodology akin to the MIRD mono-compartment method for glass microspheres was used in a phase I absorbed dose-escalation study [24]. The administered activity was calculated according to

4 $$ A\left[\mathrm{GBq}\right]=\frac{\mathrm{Liver}\ \mathrm{dose}\left[\mathrm{Gy}\right]\times {M}_{\mathrm{liver}}\left[\mathrm{kg}\right]}{15.9\ \left[\mathrm{J}/\mathrm{GBq}\right]}, $$

where the liver mass was determined on contrast-enhanced CT. The absorbed dose was escalated from 20 to 80 Gy in four steps. The maximum tolerated absorbed dose was established to be 60 Gy.

#### Limitations of current methods

An obvious limitation of these methods is that the actual spatial dose distribution of an individual patient is neglected. In general, these methods seek to prevent overdosing to the parenchyma (and lungs), minimizing the occurrence of radioembolization-induced liver disease [25–27]. As a consequence, the resultant prescribed activities are likely curbed by toxicity limitations of the most vulnerable patients and the occurrence of patients with a highly unfavorable absorbed dose distribution. This is thought to result in under-dosing in some patients [28–30].

**Fig. 1:**
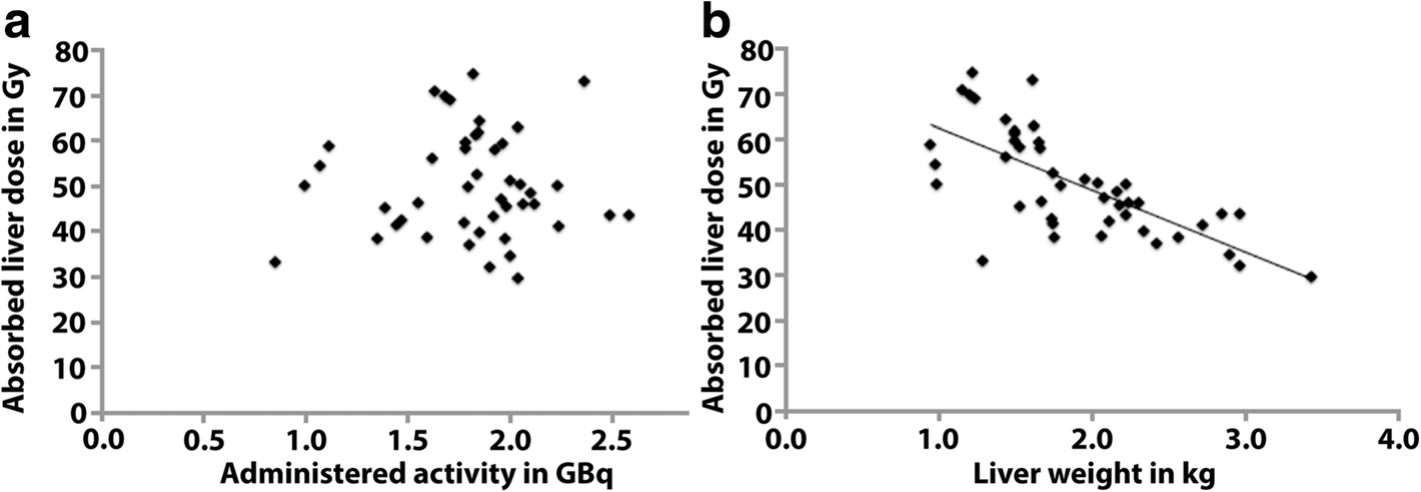
Adapted from [27]. Absorbed dose to the whole liver was not correlated to the administered activity (**a**). However, liver weight was negatively correlated with whole liver absorbed dose (*r* = − 0.723, *P* < 0.001), leading to patients with small liver being relatively over-dosed and patients with larger liver under-dosed (**b**)

For the BSA method, an added limitation is that the estimated liver volume is based on a healthy population. As such, this relation might not hold for patients with liver tumors. Indeed, it has been shown that absorbed liver dose does not correlate with prescribed activity using the BSA method [31]. This results in patients with relatively small livers that are more likely to be overdosed and patients with larger livers are more likely to be under-dosed (see Fig. 1) [29, 31, 32]. An illustration of this is given in [31] where, based on the BSA method, a patient received 1.82 GBq (BSA 1.78 m^2^, tumor involvement 15%), resulting in a high liver absorbed dose of 74.7 Gy, due to its relatively low mass of 1.22 kg. In the same study, another patient received a similar activity of 1.85 GBq (BSA 1.50 m^2^, tumor involvement 45%), but that patient had a larger liver of 2.33 kg, resulting in a much lower average liver absorbed dose of 39.7 Gy. Furthermore, there are currently no guidelines regarding activity prescription after prior resection [33].

### Multi-compartment dosimetry

A different approach to activity prescription from the homogenous, single compartment models of the BSA and MIRD mono-compartment methods is the partition model (PM). It postulates three compartments with potentially different activity uptakes: tumor, normal liver, and lung tissue [18]. As such, it allows for the selection of a prescribed activity that maximizes the absorbed dose to the tumor tissue, while not exceeding toxicity thresholds for the other two compartments. The expected activities in each compartment are usually based on the distribution of ^99m^Tc-MAA on the safety scan. However, there is some discussion in the literature about the predictive value of these particles for the subsequent ^90^Y microsphere distribution [34–37].

The respective compartments are usually segmented on an anatomical imaging modality (e.g., contrast-enhanced CT) or a functional modality (e.g., SPECT thresholding) and registered to the reconstructed ^99m^Tc-MAA distribution. The activity distribution over the compartments is described by the tumor-to-normal tissue ratio (TN ratio), expressed as

5 $$ \mathrm{TN}=\frac{\raisebox{1ex}{${A}_T\left[\mathrm{MBq}\right]$}\!\left/ \!\raisebox{-1ex}{${M}_T\left[\mathrm{kg}\right]$}\right.}{\raisebox{1ex}{${A}_{\mathrm{NL}}\left[\mathrm{MBq}\right]$}\!\left/ \!\raisebox{-1ex}{${M}_{\mathrm{NL}}\left[\mathrm{kg}\right]$}\right.}, $$

where *A* and *M* indicate the activity in and the mass of the tumor (*T*) and normal liver tissue (*NL*) compartments.

Using some algebra, the following relation can be derived for the prescribed activity, given a certain TN ratio, LSF and compartment masses [38]:

6 $$ A\left[\mathrm{GBq}\right]={D}_{\mathrm{NL}}\left[\mathrm{Gy}\right]\frac{\mathrm{TN}\times {M}_T\left[\mathrm{kg}\right]+{M}_{\mathrm{NL}}\left[\mathrm{kg}\right]}{50\left[\mathrm{J}/\mathrm{GBq}\right]\times \left(1-\mathrm{LSF}\right)}, $$

where *D*_NL_ indicates the absorbed dose to the parenchyma. Implicit in this equation is the assumption that dose is deposited locally in the compartment that contains the activity, which is a simplification. This is discussed in further detail in the section “Dosimetric models.”

Multi-compartment dosimetry is claimed to be more ‘scientifically sound’ than the BSA-based or MIRD mono-compartment method [29]. However, besides being more labor intensive to work with in clinical practice, there are several technical caveats to using the PM.

#### Different methods to calculate TN ratio

When multiple lesions are present, each may have a different microsphere uptake, leading to errors in the subsequent individual tumor absorbed dose estimates, due to averaging of the TN ratio. Mikell et al. have shown this effect by comparing the silver standard Monte Carlo-based dose estimates with the MIRD mono-compartment based dosimetry and the PM model in realistic patient data [39]. In the case of multiple tumors, there can be large discrepancies between the methods for the estimated tumor absorbed dose. For example, the variability between PM-based and Monte Carlo-based tumor absorbed dose estimates was higher by a factor five in cases where there were multiple tumors present, compared to single tumor samples.

However, there is currently no consensus on how to calculate the TN ratios for individual tumors for the use in the PM model. Some authors use the entire normal liver volume for this calculation [40], whilst others opt for a smaller sample volume, placed near the tumor-of-interest [36]. Although this simplification makes the use of the PM model more feasible in clinical practice, it also inevitably leads to larger uncertainty (≈ 2.5×) in the TN ratio estimations when the microspheres are not strictly homogenously distributed in the healthy liver tissue [39].

#### Definition of compartments on anatomical imaging

When diagnostic (contrast-enhanced) CT or MRI is used for the delineation of the tumor compartments, these delineations subsequently need to be transferred to the SPECT/CT reconstructions. This can be achieved by copying the volume-of-interest (VOI) delineation to the SPECT/CT data or by using (non-rigid) coregistration. However, mismatches are likely to occur, causing a misalignment between the anatomical delineations and the SPECT reconstruction. A common cause is differences in patient positioning between both anatomical scans. For instance, different arm positioning (above the head vs. lying next to the body) or body position (e.g., different placement on the table).

Another issue for coregistration is breathing during the CT acquisition. The acquisition of the liver volume is usually much faster than an entire respiratory period, resulting in a ‘snapshot’ of a random respiratory phase. As the SPECT or PET activity reconstruction is a superposition of all respiratory phases, this can result in mismatches of > 1 cm between the anatomical delineations and the reconstructed activity [41]. Using CTs acquired during breath-hold for coregistration might mitigate this effect, but breath-holds are shown to have a limited reproducibility between acquisitions, resulting in different relative respiratory states between scans [42]. A viable solution might be the use of so-called ‘time-averaged 3D mid-position CT scans’ in this context, often used in radiotherapy [43].

Besides leading to mismatches in coregistration, respiratory motion also results in activity reconstructions that are ‘smeared out.’ This leads to an underestimation of the local activity concentration, especially in tumor tissue, which has a smaller volume compared to the motion amplitude than the background compartment. The effect of motion blurring is well-known in general [44, 45], but the impact of respiratory motion in the context of radioembolization has recently been shown for both PET [46] and SPECT [47].

Furthermore, defining the boundaries of the tumor compartment on anatomical modalities may be non-trivial in the case of morphologically diffuse or infiltrative tumors [29]. Tumors with substantial necrosis pose a similar problem. A possible solution to this might be the use of FDG PET for the demarcation of vital tumor tissue in the case of FDG-avid tumors.

#### Definition of compartments using physiological information

**Fig. 2:**
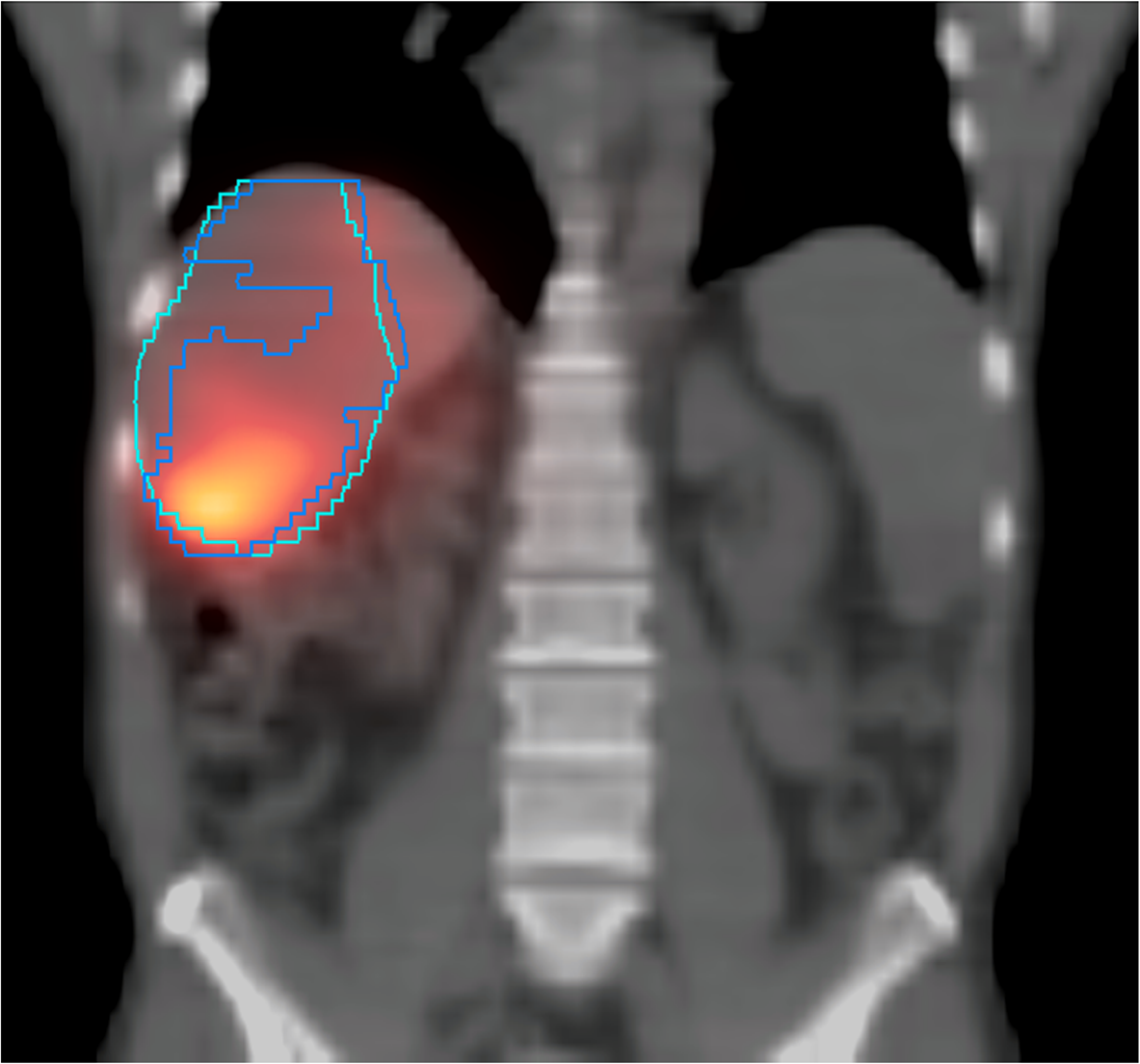
Exemplar case where a VOI delineation based on SPECT thresholding only (blue contour) does not match the CT-based anatomical tumor definition (teal contour). The mismatch results in a difference in tumor volume and mean tumor uptake

Similarly, the uptake information in SPECT reconstructions (e.g., when using MAA for absorbed dose prediction) could be used to indicate vital tumors. However, as delineations drawn directly on SPECT will generally result in errors in the estimated volume [48–50] (Fig. 2), Garin et al. have developed a hybrid method in which the SPECT reconstruction and CT information are presented in conjunction, integrating functional and anatomical information and aiding manual delineation [51, 52]. This has been shown to work well for both phantom and patient studies, in which anatomical borders are readily discernable. However, this type of method does not have a well-defined contouring guideline, which reduces reproducibility.

A more fundamental approach to this segmentation problem was proposed in a study by Lam et al. [53], in which directly after the normal ^99m^Tc-MAA SPECT scan, the participating patients were injected with ^99m^Tc-sulfur colloid (SC) and another SPECT was acquired after 5 min. This compound specifically accumulates in functional (non-tumor) liver tissue and as such will act as a negative template for the tumor compartments. By taking the difference between the MAA and SC SPECT reconstructions, voxel maps for healthy parenchyma and tumor tissue are automatically obtained, providing a ‘physiology-based segmentation.’

### Voxel-based dosimetry

In voxel-based dosimetry, the reconstructed voxel is taken as the smallest independent spatial unit for activity. This allows for the expression of (estimated) absorbed dose gradients and non-homogeneities on a small spatial scale, somewhat similar to external beam radiotherapy (EBRT). This contrasts with multi-compartment models, where absorbed dose estimates are averaged over each compartment. By including this spatial dimension, voxel-based dosimetry potentially provides a link to the rich EBRT literature on dose-effect relationships, which could potentially be used for both therapy planning and post-therapy outcome assessment. However, in contrast to image-guided absorbed dose planning for EBRT, voxel-based dosimetry for radioembolization is based on nuclear medicine images, which are generally noisy and of low resolution, prohibiting a direct translation of EBRT concepts to the radioembolization paradigm.

#### Using spatial dose information

To aid assessment and comparison between individual cases, the spatial dose information can be combined into a (cumulative) dose-volume histogram (cDVH). These graphs express the fraction of the total VOI (be it a tumor, normal tissue, or entire liver) receiving a certain minimum absorbed dose. This expresses in a single graph how the absorbed dose is distributed over the volume (Fig. 4). The concept of cDVHs also enables the introduction of spatially dependent measures of absorbed dose such as *D*_70_ (minimum absorbed dose to 70% of VOI) and *V*_100_ (percentage of VOI receiving at least 100 Gy) that might be expected to be good predictors of treatment effect [54, 55]. For example, it is clear from Fig. 4a that the blue absorbed dose distribution clearly delivers a higher absorbed dose to more of the tumor volume (or conversely, red is less toxic when this is a cDVH of normal tissue). These metrics are widely used for the comparison between EBRT plans and are gaining some traction within the radioembolization dosimetry community to help better explain clinical outcomes [54, 56–59] (see “Dose-effect relationships” section).

Due to the typical heterogeneous distribution of the microspheres, comparing cDVHs of, for instance, two patients is not always trivial (as is the case in Fig. 4a). Ambiguity can occur and an example of such a case is shown in Fig. 4b, where the cDVH curves are crossing. In such cases, it is completely dependent on the specific organ (e.g., parallel organ or not) which cDVH would lead to the highest tumor kill or least amount of toxicity.

**Fig. 3:**
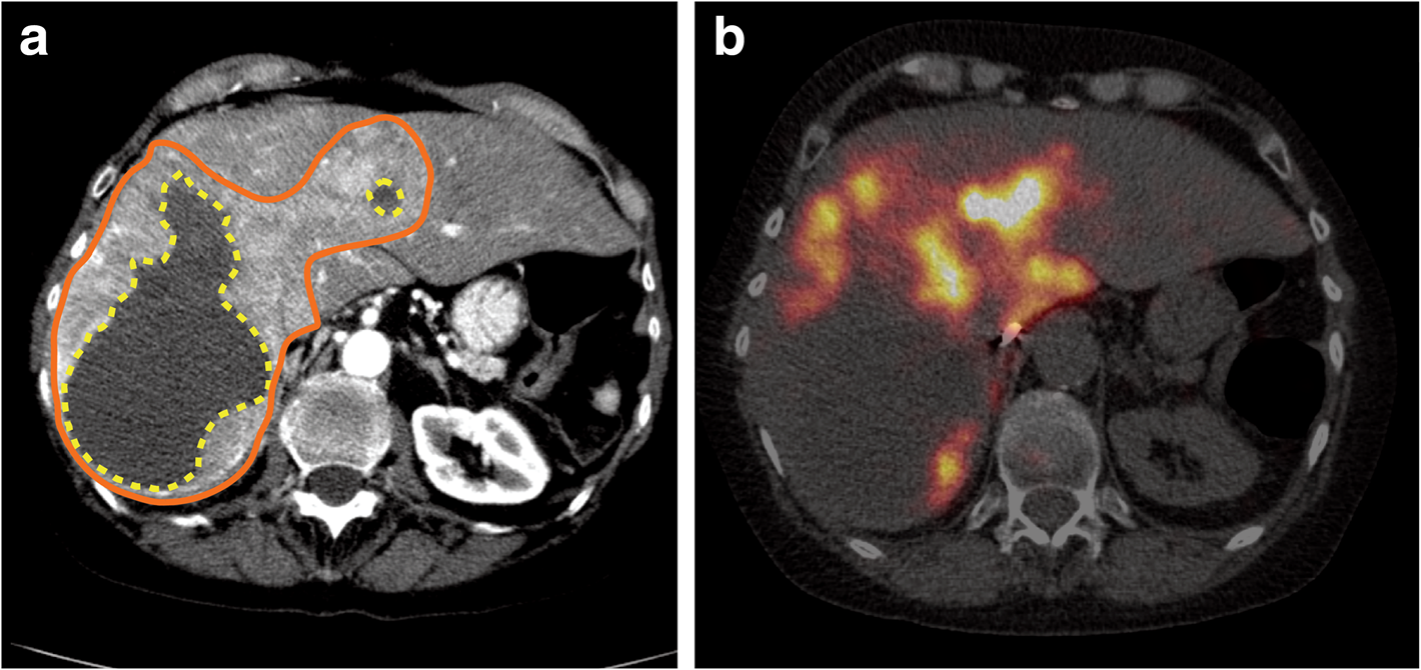
Example of a large neuroendocrine tumor, which was treated with glass microspheres. Activity was prescribed according to the MIRD mono-compartment method to reach 120 Gy. According to the PM model, the average absorbed dose to the tumor was 150 Gy. The patient has shown no response after treatment (RECIST, mRECIST, and EASL). The contrast-enhanced CT shows the tumor as a large enhanced area (orange solid line) and necrosis (yellow dotted line) (**a**). A strong absorbed dose inhomogeneity can be observed (**b**). Voxel-based dosimetry and radiobiological models may account for such absorbed dose inhomogeneities

This ambiguity is a well-known phenomenon in EBRT, and efforts have been undertaken to create radiobiological models that aim to quantify the biological effect of any treatment plan and enable the comparison of plans based on expected outcome [60]. The premise of most of these models is that an irradiated tumor exhibits a binary response (control or survival), which is determined by the surviving fraction (SF) of a population of cells after irradiation. This SF is modeled as a function of absorbed dose and may include any additional clinically relevant parameters, such as repopulation between treatments, clonogen radioresistance, and dose rate effects. Subsequently, the parameters of these models are retrospectively fitted on clinical data and can then be used to predict treatment outcome. As such, these radiobiological models provide a link between physical quantities such as spatial dose distribution and expected clinical outcome. The potential importance of radiobiological modeling is illustrated with a clinical example Fig. 3.

Importantly, two such models have been adapted from EBRT for the context of radioembolization [56, 61]. First, the effect of dose rate and cell repair mechanisms can be modeled with the biologically effective dose (BED), such that ln(SF) =  − *α* BED. BED can be calculated for a unit volume *i* (e.g. a voxel) according to

7 $$ \mathrm{BE}{\mathrm{D}}_i={D}_i\left(1+\frac{D_i\cdotp {T}_{\mathrm{rep}}}{\left({T}_{\mathrm{rep}}+{T}_{\mathrm{phys}}\right)\cdotp \alpha /\beta}\right), $$

with *D*_*i*_ the locally absorbed dose, *T*_rep_ and *T*_phys_ the halftimes for cell repair after damage and the physical halftime of ^90^Y, respectively. *α* and *β* denote the so-called intrinsic radio sensitivity and potential sparing capacity [62].

**Fig. 4:**
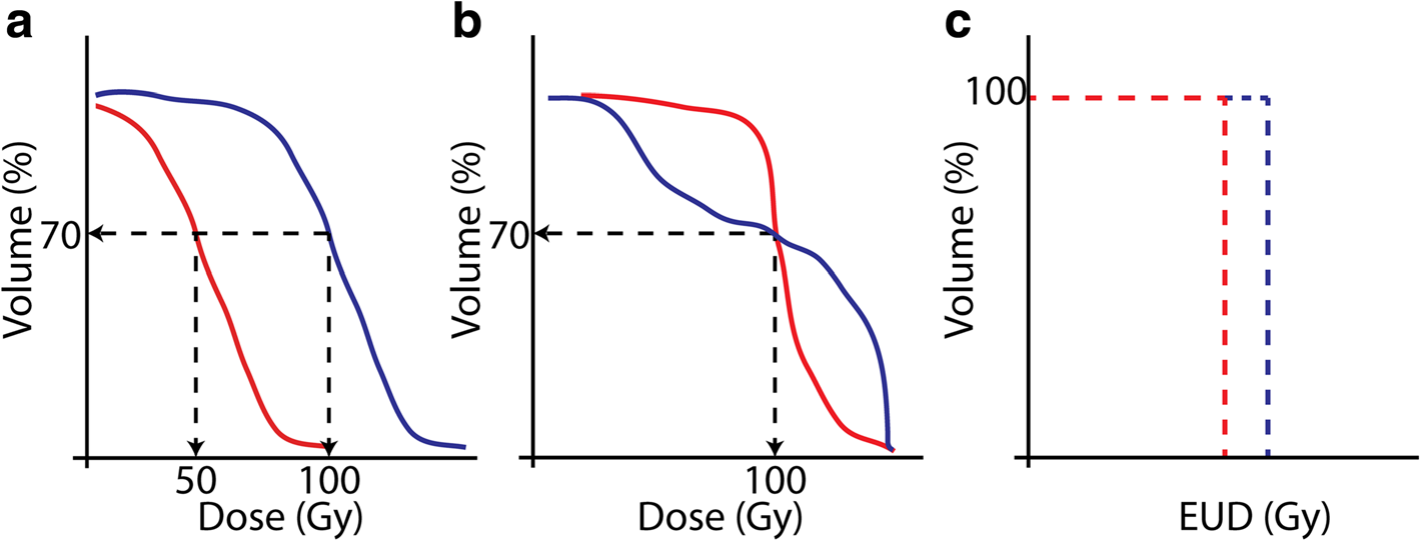
Hypothetical cDVHs illustrating key concepts in voxel-based dosimetry which may be used for outcome prediction. In panel (**a**) the situation of the red absorbed dose distribution may be expected to have a smaller impact on the tissue under consideration (less toxic or less tumor kill). This is also reflected in the *D*_70_ and *V*_100_ being lower for the red than that for the blue curve. Due to highly heterogeneous absorbed dose distributions, which is typical for radioembolization, two different cases with cDVHs as depicted in panel **b** might occur. Which of these cDVHs may be expected to have a larger effect on the tissue, is ambiguous (same *D*_70_ and *V*_100_) and might depend on the tissue type. **c** Depicts the hypothetical differences in equivalent uniform doses (EUD), derived from the situation in panel **b**, potentially resolving the ambiguity

Furthermore, spatial non-uniformities can be normalized to a single number, called equivalent uniform biologically effective dose (EUBED), see also Fig. 4c. This number is the same for different absorbed dose distributions that have the same biological effect [63]. EUBED can be defined as

8 $$ \mathrm{EUBED}=-\frac{1}{\alpha}\ln \left(\frac{\sum_{\mathrm{i}}{e}^{-\alpha {\mathrm{BED}}_i}}{n_{\mathrm{voxel}}}\right), $$

Where *α* is the radiosensitivity (1/Gy) of the local tissue, *n*_voxel_ is the number of voxels of the current VOI, and *i* denotes the voxel index [61]. A reasonable simplification for radioembolization is to neglect quadratic effects (i.e., *β* = 0) and BED, in which case BED_*i*_ is substituted with *D*_*i*_ in Eq. 8, which then yields equivalent uniform dose (EUD).

In theory, this approach will aid physicians to optimally weigh risks and benefits of an individual absorbed dose distribution, as clinical outcomes can be linked to a single number such as BED and EUBED. However, the existence and robustness of such dose-effect relationships in the context of radioembolization are currently still under investigation.

### Dose-effect relationships

There is an increasing literature on dose-effect relationships in radioembolization that utilizes advanced dosimetry. An overview of recent papers that (implicitly) estimate tumor control probability (TCP) and/or non-tumor complication probability (NTCP) is given in Table 1. The search for the combination of tumor type, outcome measure, dosimetric model, and imaging modality that yields the best predictive power is very early stage. Consequently, there is a wide variety in each of these properties amongst these studies, resulting in diffuse optimal absorbed dose limits for both liver complications (~ 50–97 Gy) and tumor control (~ 50–560 Gy). Four major factors are hypothesized to contribute to this: differences in response measures, absorbed dose calculations, microsphere type, scan modality (including acquisition and reconstruction settings), and tumor type.

#### Response measures

In these studies, tumor response is assessed according to RECIST, mRECIST, vRECIST, EASL, densitometric change [64], change in total lesion glycolysis (TLG), or standardized uptake value (SUV). What is considered a complete response, partial response, stable disease, or progressive disease differs significantly between these measures [65]. For example, the RECIST criteria are sensitive to changes in tumor size, whereas TLG expresses (changes in) total glycolysis (tumor volume times mean SUV over the VOI). Consequently, minimum absorbed dose estimates that lead to tumor response are different between criteria. Although some attempts have been made to directly compare some of these methods [66], the use of such a variety of methods makes comparing these data non-trivial, if not impossible. As the most relevant clinical outcomes are overall survival and progression-free survival, it is important to establish which of the reported proxies is the most predictive of survival [67]. This may result in disease-specific outcome measures (e.g., EASL or mRECIST for hepatocellular carcinoma and RECIST or TLG for metastatic colorectal cancer).

Some studies that are reported in Table 1 incorporate either metabolism-based (functional) masks from a previous FDG PET [55] or, for example, the *D*_70_ measure [54]. However, most studies calculate the average absorbed dose to the tumor. This may disregard the existence of necrotic volumes and, more generally, absorbed dose heterogeneity.

#### Absorbed dose calculations

Which absorbed dose calculation method best reflects the underlying radiobiological processes in the entire patient population remains an open question. Theoretically, applying EUD and/or BED-based models should be best suited to naturally incorporate differences in specific activity and absorbed dose heterogeneity in a tissue, as described above. But clinically, a clear advantage over average absorbed dose to the tumor is yet to be found [56, 61, 68, 69]. The authors suggest this might be linked to the outcome measure being too crudely categorized [61]. Another central finding in these studies, however, is that the apparent radio sensitivities (*α* and *β* in Eq. 7) of both the tumor and hepatic tissues in radioembolization are an order-of-magnitude lower than what is found in the EBRT setting, even when correcting for absorbed dose inhomogeneities [61]. Moreover, a significant difference between the absorbed dose needed to reach TCP(50%) for glass and for resin microspheres has been found [61, 68]. In conclusion, the values of the relevant parameters in the radiobiological models have not been well established for radioembolization. They may be specific to the type of microsphere and it can even be expected to be different between ^90^Y and ^166^Ho-based microspheres. These uncertainties have a direct impact on the determination of BED and EUD.

#### Micro-distribution

A possible explanation for the differences found between glass and resin microsphere dose-effect relationships is the potential difference in micro-distribution.

One of the first papers on in vivo microsphere distribution was by Fox et al. [70]. Using a beta-probe, they showed that the activity pattern on a sub-centimeter scale was highly heterogeneous. Later, Yorke et al. [71] used a combination of computer simulations and biopsy samples to try to find an explanation for the clinically observed lack in normal liver complications using glass microspheres at absorbed dose levels that are known to cause complications in EBRT and found that absorbed dose heterogeneity is sufficient to explain this incongruity.

**Fig. 5:**
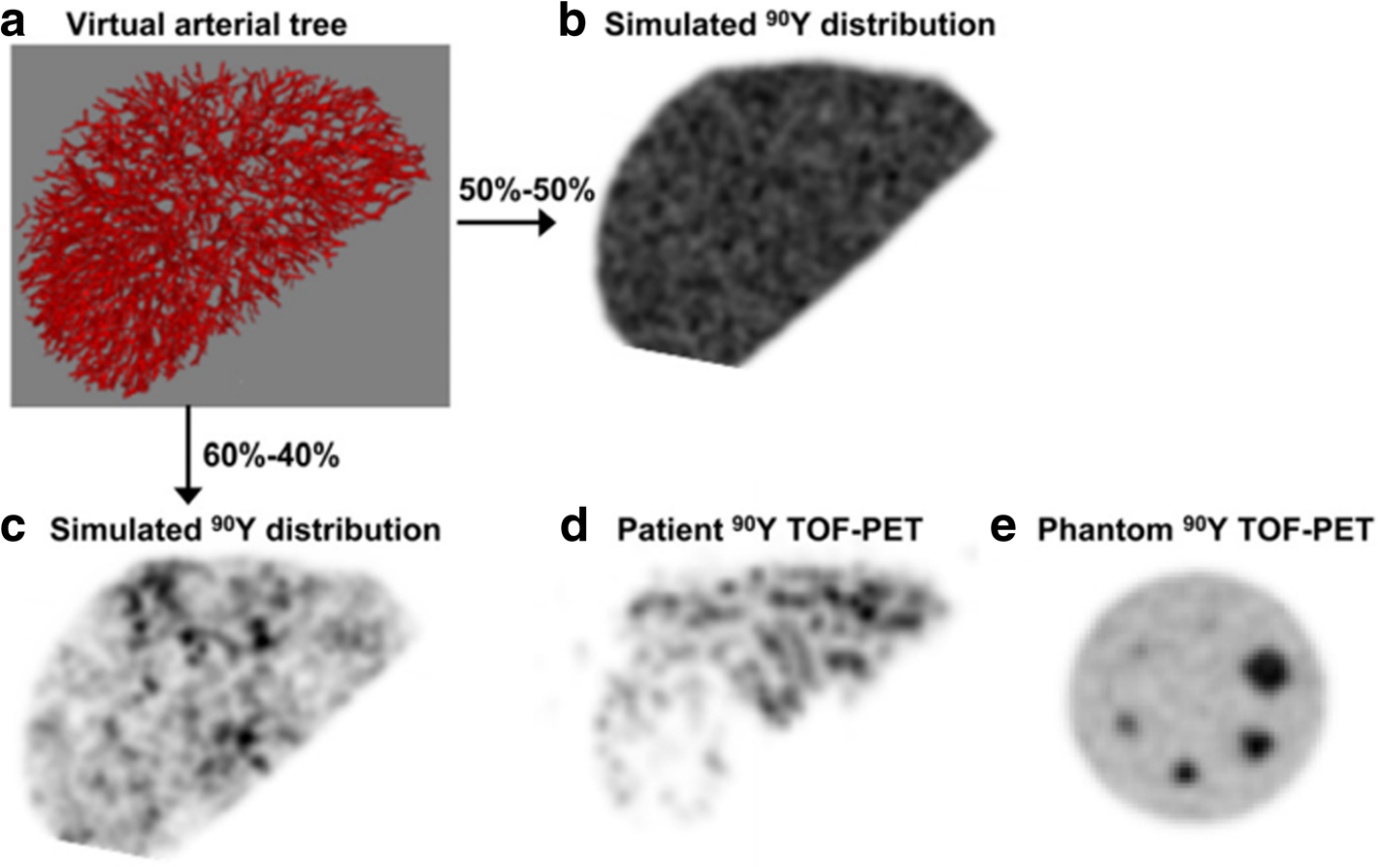
Simulated arterial tree (**a**) and subsequently simulated microsphere distribution after flow through the arterial tree (**b**, **c**), which explains PET ‘mottled’ look often found in patients (**d**) but not in phantom scans (**e**). This research was originally published in JNM [72]. Copyright by the Society of Nuclear Medicine and Molecular Imaging, Inc.

More recently, Walrand et al. performed a simulation study of normal liver tissue, finding that the relatively low number of injected glass microspheres results in non-uniform trapping in the terminal portal artery, resulting in tissue volumes receiving sub-lethal absorbed doses. This would both explain the relatively low toxicity per Gray of glass, relative to resin microsphere and the granularity observed in post-treatment ^90^Y PET (see Fig. 5) [72].

This conclusion was seemingly contradicted in an elaborate histological study by Högberg et al., who found that a higher concentration of microspheres (i.e., in the case of resin microspheres) leads to a higher tendency to form clusters, especially in the larger (upstream) arterioles, resulting in a more non-uniform absorbed dose distribution in the liver parenchyma [73]. According to these authors, this apparent contradiction stems from the fact that Walrand et al. only assumed microsphere trapping in the terminal branches of the infused artery. In a subsequent simulation study, Högberg et al. were able to replicate their histological findings in a mathematical model. This places further emphasis on the importance of the geometry of the arterial tree and (local) microsphere concentration as drivers for microsphere distribution inhomogeneity [74]. These models, however, predict cluster propensity as a function of arteriole generation (branch number) and lack further spatial information. Consequently, the authors conclude that the micro-scale clusterings they observed in itself might not (fully) explain the observed macroscopic inhomogeneities, as measured by non-invasive imaging [73].

**Fig. 6:**
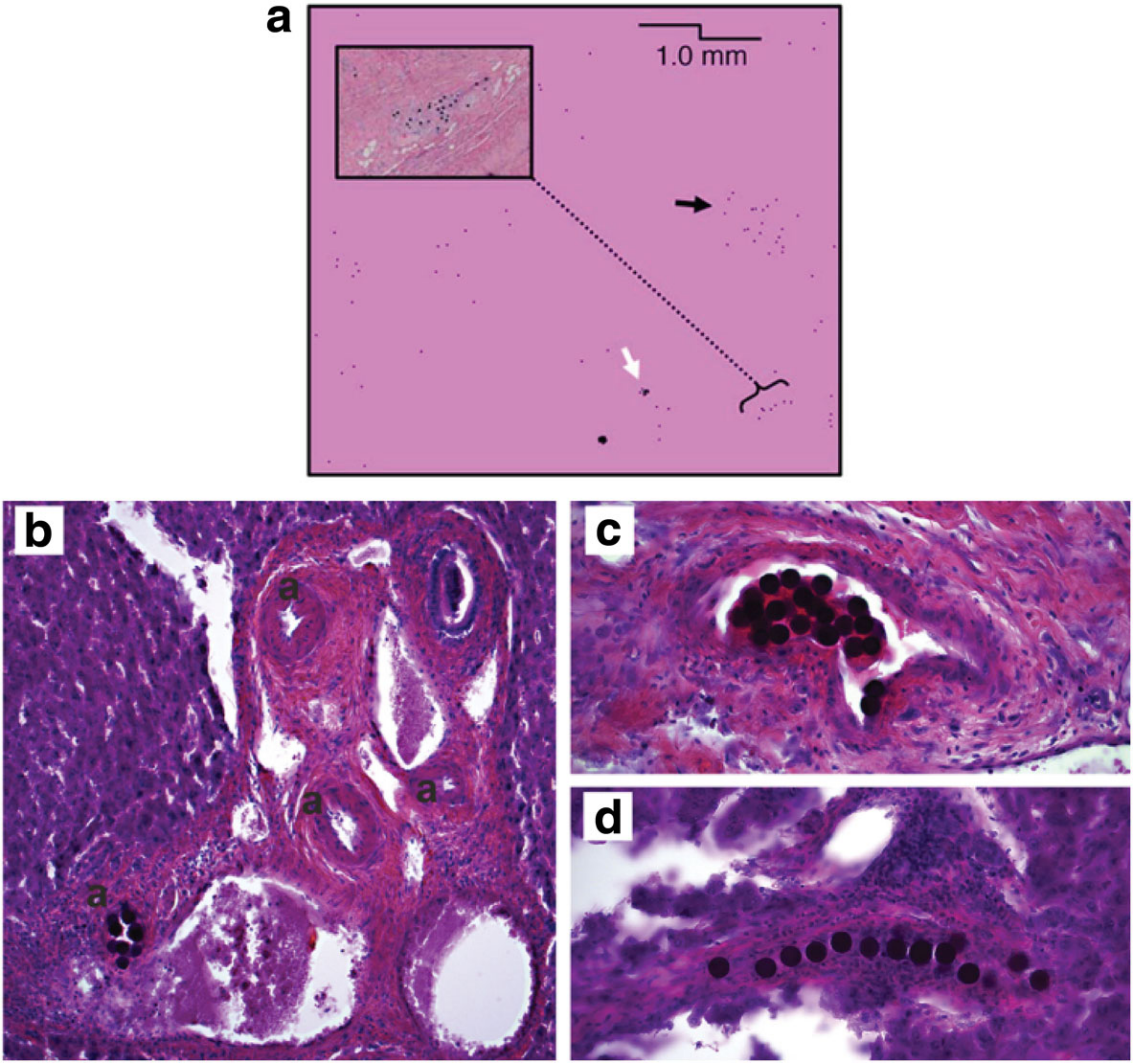
**a** Small clusters (white arrow) and large clusters (black arrow) are apparent in the Monte Carlo simulations by Pasciak. These simulated distributions seem to be consistent with the histological findings of (amongst others) Högberg (**b**, **c**, **d**). Panel **a** was originally published in JNM [75]. Copyright by the Society of Nuclear Medicine and Molecular Imaging, Inc. Other panels are adapted from [74]

Pasciak et al. tried to bridge the gap between micro- and macro-scale tumor dosimetry by using Monte Carlo-based estimations of microsphere micro-distributions, given a ^90^Y PET reconstruction of a patient [75]. These microsphere micro-distributions are simulated by drawing properties such as cluster propensity and distance from probability density functions that were constructed from histological data [76]. This resulted in realistic structures (Fig. 6) such as clusters and strings of microspheres. Crucially, it provides a plausible link between the observations in macro- and microdosimetry.

### Quantitative image reconstruction

Besides the abovementioned factors concerning dosimetry, differences in outcome between these studies may in part also be explained by the wide range in technical scan parameters used and the measurement variance inherent to nuclear medicine images. An overview of the current topics in nuclear image acquisition and reconstruction is therefore desired.

In quantitative image reconstruction, all relevant interactions between the radionuclide, the patient, and the imaging system need to be accounted for during reconstruction. The current state-of-the-art consists of iterative reconstruction algorithms that incorporate models for all such image-degrading effects (e.g., attenuation, scatter, nuclide decay, detector uniformity) [77–79].

From its inception, PET was considered a quantitative modality, in contrast to SPECT. This is due to the high signal-to-noise ratio of PET and the relative simplicity of the physics of coincidences, which enables a straight-forward method for attenuation correction. This was available in early generations of PET scanners. However, with the advent of inherently coregistered CT in SPECT/CT, attenuation and scatter correction are now common practice and some authors have claimed that modern clinical SPECT systems can now be considered quantitative as well [80, 81]. Furthermore, vendors are currently implementing calibration routines and inherently quantitative reconstruction software in their machines [82, 83], which enables the dissemination of absolute activity quantification into clinical practice for both modalities.

#### Post-therapy imaging

In radioembolization, treatment success can be assessed with a post-therapy scan, either with bremsstrahlung SPECT/CT (bSPECT) or PET/CT (^90^Y PET).

##### Bremsstrahlung SPECT/CT—the relevance of physics modeling

Post-therapy assessment with ^90^Y may be performed by bremsstrahlung imaging. This is different from mono-energetic emitters (such as ^99m^Tc) in that ^90^Y produces a broad and continuous energy spectrum without a photopeak. Secondly, the high flux of bremsstrahlung photons on a gamma camera will result in significant dead time, if not managed correctly (count rate linearity was estimated up-to 7.5 GBq for ^90^Y and 1.5 GBq for ^166^Ho [84]). Therefore, ^90^Y image quantitation using a gamma camera was recognized early on as being non-trivial [85]. With the advent of advanced iterative reconstruction techniques that enable advanced physics modeling, several quantitative reconstruction methods have been proposed in the literature. Most of these methods utilize some kind of Monte Carlo modeling of the imaging process. Rong et al. achieved quantitation errors between − 1.6 and 11.9% for a phantom experiment by modeling all relevant energy-dependent image degrading effects [86]. Elschot et al. incorporated Monte Carlo simulations of photon-tissue interactions directly within the iterative reconstruction loop, increasing image contrast and activity recovery significantly (over 80% for non-small spheres), relative to a reference clinical reconstruction algorithm [87]. Minarik et al. performed a similar study, using the SIMIND code and achieved a quantification error around + 8.5% [88].

However, these methods rely on advanced Monte Carlo techniques, which are currently not easily accessible for institutions without a medical physics team that has extensive experience with these methods. Consequently, the reported accuracies will be significantly worse in normal clinical practice.

##### ^90^Y PET—the impact of machine and reconstruction parameters

^90^Y can also be imaged using PET. However, as ^90^Y only has a minute positron branching ratio (~ 32 ppm) and the detectors were expected to be saturated from the bremsstrahlung photons (which was later demonstrated to be false), for a long time, PET was not considered a feasible modality for post-therapy imaging. The earliest in vivo demonstration of the feasibility of ^90^Y PET imaging was delivered by Lhommel et al. in 2009 [89] using a time-of-flight (TOF) PET scanner and an additional copper ring inserted in the gantry to prevent detector saturation. Later, the feasibility of ^90^Y PET with Lutetium oxyorthosilicate (LSO) crystals in a scanner without TOF capability was demonstrated [90].

These initial proofs-of-concept were followed by studies that corroborated the quantitative reconstruction capabilities of ^90^Y PET, using clinically available methods [91–94] which were applied to clinical data [54, 95–97]. However, the very high contribution of randoms (> 90%) due to bremsstrahlung in combination with the very low coincidence count statistics was expected to impact random coincidence estimation, scatter correction, and consequently, image quality. An elaborate study by Carlier et al. has shown that the effect of these phenomena on bias, variability, and detectability of hotspots is minor. The use of correct point spread function (PSF) modeling and TOF reconstruction kept background variability and noise at acceptable levels [98]. This was further corroborated in a fully Monte Carlo-based simulation in which ^90^Y quantitation is compared to that of ^18^F [99]. It was found that, relative to ^18^F, the image quality was only slightly poorer in ^90^Y for a similar positron emission rate. Furthermore, image quality was not strongly linked to any particular physical effect or reconstruction step. This led to the conclusion that adding ^90^Y-specific models to the PET imaging process is not needed. Furthermore, Van Elmbt et al. have shown that systems based on modern crystals (post-BGO) can be used for ^90^Y dosimetry [100]. Since then, ^90^Y image quantification has also been shown to be possible in PET/MRI [101] and solid-state digital PET/CT [94].

For clinical ^18^FDG PET, the importance of homogenization of acquisition and reconstruction settings over centers to allow pooling and comparison of data sets is well-recognized and has resulted in the EARL guidelines and accreditation program [102]. A similar initial attempt for ^90^Y PET has been made in the form of the QUEST study, showing that ^90^Y PET-based dosimetry should be reproducible across scanners and centers, as long as TOF-capabilities are available [103].

Together, these studies show that PET-based ^90^Y quantitative imaging is feasible, robust, and straight-forward to implement in clinical practice when a reasonably modern PET system with TOF-capabilities is available. This is in contrast to bSPECT/CT, for which no sufficiently accurate reconstruction methods are currently available for general clinical use.

##### ^90^Y PET vs. bSPECT

In a direct comparison between bSPECT/CT and ^90^Y PET/CT, the latter is found to have a higher resolution and less scatter in patient studies and several case series [104, 105]. In a quantitative direct comparison between a state-of-the-art clinical bSPECT/CT reconstruction algorithm and clinical PET/CT reconstruction protocols, the superior contrast, detectability, and absorbed dose estimates of PET were demonstrated [106]. However, this comes at the price of a relatively long scan duration in the case of ^90^Y PET, which is 15 to 20 min per bed position. When advanced photon-tissue and photon-detector interactions were modeled with a Monte Carlo-based SPECT/CT reconstructor, image contrast improved substantially and was in some cases (in larger hot spots) higher than in PET/CT [87].

In general, it should be noted that currently there is no standardized approach for post-therapy imaging in terms of acquisition and reconstruction settings and there may exist some systematic biases between the various approaches of different groups, even within the same modality. As a consequence, interpreting and comparing dosimetric results between different groups should be done with caution. However, in general, ^90^Y PET is currently superior to clinical bSPECT/CT in terms of resolution, accuracy, and the clinical availability of accurate reconstruction methods for dosimetry.

##### MR and CT for ^166^Ho

In contrast to ^90^Y-based microspheres, ^166^Ho does emit photons with discrete energies that are directly detectable with a gamma camera. Furthermore, it is a paramagnetic element, enabling the visualization with MRI, and it has a very high X-ray attenuation, resulting in good contrast on CT [107, 108]. A quantitative SPECT/CT reconstruction using advanced Monte Carlo-based techniques has been developed [109] (achieving contrast recovery of over 80% in non-small NEMA spheres), as is a hybrid method to correct for photon down-scatter from bremsstrahlung and higher energy photons [110]. In a direct comparison between SPECT- and MR-based quantification [108], both modalities are found to be suited for peri-therapy dosimetry [111].

### Dosimetric models

With quantitative imaging, the physical quantity activity (i.e., Becquerel or Curie) of the isotope distributed in space is estimated. However, especially in the case of radionuclide therapies, the process of interest is not the activity per se, but rather the subsequent dose absorption by the surrounding tissue (in Gray), as a result of high energy particles (betas and photons) emitted in the process of decay. This process of dose absorption is what causes the tumor kill and constitutes a rather complex interaction, which depends both on the tissue and the specific emissions from the isotope.

If the isotope distribution is known exactly, the most comprehensive and precise estimations of absorbed dose are achieved through Monte Carlo simulations of all relevant interactions between the high energy particles and the healthy or tumor tissue. Popular codes include the EGSnrc code [112], MCNP [113], FLUKA [114], and the GATE extension of GEANT [115].

However, these types of simulations are rather complex and time-consuming. Furthermore, the liver is a rather homogenous medium in terms of dose absorption at energies typical for radioembolization. Therefore, a frequently used method to speed up these calculations is by pre-calculating a dose point-kernel (DPK) or dose voxel-kernel (DVK), which is energy absorption in a homogeneous medium around a point source or a voxel source, respectively. Then, a convolution of the true activity distribution with the DPK/DVK will result in an accurate absorbed dose estimation for a homogeneous medium. This kernel can also be scaled to different local tissue densities [116].

The largest contribution to the total absorbed dose comes from the emitted beta particles. The maximal range for ^90^Y betas in tissue is 1.2 cm (0.9 cm for ^166^Ho), which is in the same order of magnitude as the resolution of both SPECT and PET. This implies that most of the energy is deposited within the voxel of origin. Consequently, a further simplification is to assume that all emitted energy is absorbed locally, which is usually called the local deposition model (LDM). In practice, this method constitutes applying an appropriate scaling factor to the voxel values of a quantitative reconstruction.

In a direct comparison of SPECT-based ^90^Y dosimetry Monte Carlo, DVK and LDM-based dosimetry are found to be nearly identical for activity which is not close to tissue inhomogeneities (e.g., liver-lung border) for the liver [117]. For lung tissue, it was necessary for the DVK method to be scaled to the lower local tissue density of the lung tissue to reach adequate results.

This lack of difference between LDM and the other models is likely to be explained by the fact that a SPECT-based reconstruction has a resolution that is in the same order as the average beta-range. This can be understood as a convolution of a ‘blurring’ kernel with a putative perfect activity distribution, obviating the need for the simulation of the beta-transport (i.e., blurring). LDM does not unnecessarily repeat this step. Indeed, Pasciak and Erwin found for ^99m^Tc-MAA SPECT reconstructions that LDM outperformed a Monte Carlo-based absorbed dose estimation, due to this effect [118]. Later, this finding was repeated in ^90^Y PET [119]. Although in most cases for PET, much of the theoretical benefit is obscured due to image noise, causing both techniques to have a similar absorbed dose uncertainty. Still, the authors recommend using the LDM in post-radioembolization ^90^Y PET dosimetry due to its accuracy and ease-of-use [119].

### Timing of dosimetry-based treatment planning

Pre- and post-treatment are not the only time points for dosimetry, as Bourgeois et al. report on intra-procedural PET/CT in a case study [120]. They used a 3-step protocol wherein the first step ^90^Y microspheres with a total activity determined by the BSA model were administered to the patient. For the second step, the patient was transferred for PET/CT imaging. The maximum absorbed dose by normal hepatic parenchyma and the average absorbed dose by the tumor were determined. For one out of the six patients in this study, the absorbed dose by the tumor was below the assumed tumoricidal absorbed dose of 100–120 Gy for HCC (the other five patients reached this threshold with the first infusion, which was based on the BSA model). For this single, undertreated patient, the third step of the protocol was performed which was a repeated infusion of ^90^Y microspheres with an optimized activity determined from the quantitative PET/CT data to reach the target tumor absorbed dose. Although the initial treatment planning was based on the suboptimal BSA method, the dosimetry based on the intra-procedural PET/CT scan allowed for activity delivery based on patient-specific physiology at the time of the procedure. The downsides of this 3-step protocol are the increased time, costs, and access to equipment and personnel.

This last disadvantage might be partly solved by imaging in the intervention room. Walrand et al. describe a camera dedicated to bremsstrahlung SPECT of ^90^Y [121]. They suggested mounting the gamma camera on a robotic arm to allow SPECT acquisition within a few minutes in the intervention room during the catheterization procedure to optimize the ^90^Y activity to inject.

Another option for imaging in the intervention room is proposed by Beijst et al. [122]. The authors propose a hybrid imaging system, consisting of an X-ray c-arm combined with gamma imaging capabilities for simultaneous real-time fluoroscopic and nuclear imaging. A slightly modified version of this prototype [123] was shown to be able to accurately estimate LSF of a ^99m^Tc-MAA scout dose in an interventional setting [124]. When this hybrid imaging modality becomes available in the angiography room, it may be possible to move towards 1-day procedures by combining scout and therapy dose in one session.

Using microspheres labeled with a paramagnetic element, like ^166^Ho, will provide contrast on MRI. It has been shown that the absorbed dose by the tumor and healthy liver can be accurately quantified using a post-treatment MRI scan [108, 112]. Since MRI provides excellent soft tissue contrast, it would be a well-suited modality for radioembolization guidance as well as evaluation of therapy. The feasibility of fully MR-guided real-time navigation of hepatic catheterization was demonstrated in an animal model [125]. Drawbacks of MR-guided radioembolization are the potentially limited availability of MR scanners and MR-compatible catheters, and guide wires and the relatively high costs.

## Discussion

Recently, several phase III trials failed to show an improvement in progression-free survival and overall survival when radioembolization with SIR-Spheres was combined with first-line treatments. A reason for this might be that the methods for activity prescription which were used in these studies (BSA and MIRD mono-compartment) are barely personalized and are geared towards safety rather than efficacy. More personalized methods (e.g., the partition model, cDVH-based methods) are available. However, there is no consensus as to what absorbed dose thresholds should be prescribed. In this manuscript, we have therefore reviewed the specific shortcomings of the current activity prescription methods and the current state-of-the-art of newer dosimetric methods and understanding of the underlying radiobiology.

Currently, there is a large range in the literature regarding dosimetric limits, both for the TCP and the NTCP (Table 1). We believe that one of the biggest drivers of these diffuse limits is the corresponding wide range in modalities, technical settings, analysis, clinical outcome measures, and relatively small sample sizes. It is therefore nearly impossible to compare data from different studies and distill a common absorbed dose limit, regardless of dosimetric method. This also highlights the importance of investigators providing clear and detailed information on the dosimetric method and analysis used in their publication. This will facilitate reproducibility and may allow for the pooling of clinical data.

With the advent of advanced iterative reconstruction techniques, image quality has improved dramatically in both PET and SPECT [80]. This is mainly due to the incorporation of models for the physics of image formation. In contrast to PET and probably owing to the more complex (underdetermined) nature of the physics in SPECT imaging, dissemination of quantitative reconstruction algorithms started only recently for SPECT [84, 126]. For more complex isotopes (e.g., ^90^Y bremsstrahlung SPECT), this is still in the research phase and vendor-supported solutions are currently not available. The same holds for more complex image-degrading effects (for both SPECT and PET) such as respiratory motion and compensation for partial volume effects. These developments are beneficial to the goal of personalized dosimetry but are currently not widely available.

However, we believe that using the currently available reconstruction techniques, reliable estimates of dosimetric limits may be established. But in order to achieve comparable results, acquisition and reconstruction settings should be standardized. An initiative similar to the EARL accreditation program for ^18^FDG PET should lead to reconstructions that are perhaps less than optimal but are at least comparable between patients and institutions, allowing for the pooling of derived dosimetric data. To illustrate this point, it was recently shown that it is feasible to get reliable absorbed dose estimates from ^99m^Tc-MAA, even if attenuation correction is lacking, using only a simple calibration [62]. This lowers the technical demands for more personalized dosimetry. For technical parameters in ^90^Y PET, the QUEST initiative may be regarded as a step in the right direction [103].

Ideally, this standardization should not be limited to technical parameters of a specific imaging modality, but should also include methods for the segmentation of compartments (or voxels of any VOI), the transferring of volumes (e.g., pre-therapy image delineations transferred to post-therapy images), the selection of relevant clinical outcome measures, and stratification by relevant clinical factors (e.g. tumor size, tumor type, baseline liver function). We believe that this standardized acquisition and analysis pipeline may solve the biggest sources of error in current comparative studies (especially multicenter ones). Furthermore, this standardized protocol can be used for prospective studies in dosimetric limits that are relevant to clinicians.

We expect that the formulation of and adherence to such a standardized protocol would be greatly aided if it is based on guidelines formulated by a panel of experts, ideally sponsored by an authoritative entity such as the EANM, SNMMI, or similar.

In order to further refine personalized dosimetry in clinical practice, new methods need to be fast in terms of scan time, labor extensive, robust, and standardized. Some examples of developments in this direction are fully data-driven respiratory motion compensation [127, 128] and fast lung absorbed dose estimation [129]. These methods have in common that they are faster, often more reliable and robust and more usable in clinical practice than existing methods. As there is a wide range of clinical parameters that have an influence on response to therapy, we believe that currently the biggest challenge for the medical physics community involved in radioembolization is not the improvement of quantitative imaging in itself, but rather the translation and dissemination of the current state-of-the-art into a usable form for the use in practical dosimetric efforts. The potential increase in clinical workload and costs associated with further refined personalized dosimetry should be weighed against the potential gains and should not a priori be considered a barrier for implementation.

Currently, many aspects of fundamental radiobiology in radioembolization are unknown. For example, the group of Chiesa et al. was unexpectedly unable to show a clear increase in predictive power for outcome when using equivalent uniform dose-based measures, as opposed to the average absorbed dose to the tumor or healthy liver tissue [61]. This illustrates that radiobiological model parameters are not well established for this modality and that the precise relation between absorbed dose non-homogeneity at both the macro (voxel) level and sub-millimeter level and tissue response is not well understood. We expect that a better understanding of radiobiology on this level will aid the establishment of a coherent account on the efficacy of radioembolization and to enable further refinements in patient selection and/or personalized dose optimization. In that sense, the combination of statistical histological data with models that bridge between micro-distribution and clinically observable macroscopic features in reconstructed data (e.g., ‘mottled look’ in 90Y PET) might provide additional insight into (deviations from) dose-response relationships in a wide population of patients and may result in a micro-scale equivalent uniform dose-metric.

An improved understanding of radiobiology may also facilitate other concepts from EBRT to be translated to the context of radioembolization. An example is fractionation, which uses tumor repopulation and oxygenation between fractions to increase the tumoricidal effect of subsequent irradiations. For radioembolization, this would mean improved tumor control for multiple vs. the current single treatment (e.g., two times 60 Gy vs. 120 Gy at once). Whether or not this effect can be exploited using radioembolization is an area of future research.

A better understanding of the dose-response relationships will lead to an improved selection of patients for which dose may be increased safely. Currently, the only available particle for treatment planning is ^99m^Tc-MAA, which might be a suboptimal predictor of the subsequent microsphere biodistribution, both in terms of LSF [15, 16] and intrahepatic distribution [35–37]. Consequently, a particle that better matches the rheology of the therapeutic microspheres is needed, if radioembolization is to become a true theranostic modality [130]. Several efforts in this direction have been undertaken [131–133]. In this context, ^166^Ho is a promising alternative in that exactly the same particle can be used for both planning and therapy. This was illustrated for the estimation of LSF [15].

Another approach is to apply dosimetry in an interventional setting [120]. For instance, following an AHASA (as high as safely attainable [8]) paradigm during infusion of the microspheres until thresholds for hepatic toxicity are reached.

Furthermore, if dosimetry is found to be sufficiently reliable, EBRT could be used after radioembolization on specific target areas that might have received a sub-optimal absorbed dose from radioembolization.

Together, these developments in homogenization, accessibility, and improved methods will ideally lead to the most personalized and optimal treatment, which we expect to result in improved overall survival and progression-free survival.

## Conclusions

A better understanding of dose-response relationships is needed for improved patient selection and dose optimization in radioembolization. To this end, standardization of acquisition, reconstruction, and analysis protocols are needed. Such an effort would greatly benefit from centrally formulated guidelines. This might enable comparison and pooling of clinical data. Disseminating advanced methods from research groups to clinical practice could prove to be useful in this respect.

## Abbreviations

^99m^Tc-MAA: 99mTc macroaggregated albumin
BED: Biologically effective dose
BSA: Body surface area
bSPECT: Bremsstrahlung SPECT
cDVH: Cumulative dose-volume histogram
Cholangio: Cholangiocarcinoma
CR: Complete response
CRC: Colorectal cancer
DPK: Dose-point kernel
DVK: Dose-voxel kernel
EBRT: External beam radiotherapy
EUD: Equivalent uniform dose
HCC: Hepatocellular carcinoma
LDM: Local deposition model
LMER: Linear mixed-effects regression model
LSF: Lung shunt fraction
NET: Neuroendocrine tumor
NTCP: Non-tumor complication probability
PM: Partition model
PR: Partial response
PSF: Point spread function
ROI: Region-of-interest
SC: Sulfur colloid
SD: Stable disease
SF: Surviving fraction
SUV: Standardized uptake value
TCP: Tumor control probability
TLG: Total lesion glycolysis
TN ratio: Tumor-to-normal tissue ratio
TOF: Time of flight
VOI: Volume-of-interest
